# Entrepreneurial Learning, Self-Efficacy, and Firm Performance: Exploring Moderating Effect of Entrepreneurial Orientation

**DOI:** 10.3389/fpsyg.2021.731628

**Published:** 2021-08-26

**Authors:** Yan Shen, Qi Wang, Danni Hua, Zhetao Zhang

**Affiliations:** ^1^Jing Hengyi School of Education, Hangzhou Normal University, Hangzhou, China; ^2^School of Management, Zhejiang University of Technology, Hangzhou, China; ^3^College of Business and Public Management, Wenzhou-Kean University, Wenzhou, China

**Keywords:** entrepreneurial learning, entrepreneurial self-efficacy, entrepreneurial orientation, firm performance, formal organizational learning, intergenerational learning, social network learning

## Abstract

Although the impact entrepreneurial learning on firm performance has attracted significant attention, a comprehensive understanding by integrating entrepreneurial orientation and individual self-efficacy remain poorly understood. We fill this void by integrating the above variables into a model and examine these relations. Findings from a sample of 411 nascent entrepreneurs support that entrepreneurial learning is positively related to firm performance, and this relationship is fully mediated by entrepreneurial self-efficacy (ESE). We also found entrepreneurial orientation strengthens the positive impact of entrepreneurial learning on ESE. The findings indicate that ESE must be in place to maximize the effect of entrepreneurial learning on performance, and entrepreneurial orientation is an important contingency in shaping entrepreneurial learning's impact on nascent entrepreneur's self-efficacy.

## Introduction

It is widely acknowledged that entrepreneurship is the vigor and vitality of economy (Cope, 2005; Jin et al., 2021; Yousaf et al., 2021) and the entrepreneur is the catalyst for entrepreneurial activity (Cope, 2005; Parker, 2013; Feng and Chen, 2020). Therefore, it is vital to research entrepreneur who represents the essence of entrepreneurship. Although more and more young graduates join the entrepreneurship boom, thousands of entrepreneurs fail each year and the new venture mortality rates remain relatively high (Klimas et al., 2021; Lattacher et al., 2021). Some statistics indicate only two-thirds of small businesses survive at least 2 years while 50% fail to sustain operations beyond their fifth year (Jawula, 2021). The startup's demise may be caused by a lack of entrepreneurial preparedness. New ventures are difficult to cope with high-level uncertainty of market environment due to lack of sufficient entrepreneurial preparedness. Fortunately, scholars have begun to emphasize that entrepreneurial learning is a key ingredient for firm survival and success, especially a new business in its early developmental stages.

Entrepreneurial learning is becoming a hot research topic at the interface between learning and the entrepreneurial context (Harrison and Leitch, 2005; Casillas et al., 2015; Liu et al., 2019). Accumulating research has explored the significant influence of entrepreneurial learning in entrepreneurship. It is noted that substantial research has been focused on entrepreneurial intention formation of young adults (Wilson et al., 2007; Hu et al., 2018; Shi et al., 2019). These studies, however, pay more attention to individuals' behavior whether to start a business, but ignore the impact of entrepreneurial learning on established new ventures. Moreover, extant research has provided insight into organizational learning of mature entrepreneurs and corporate performance (Zhao et al., 2011; Real et al., 2014), but little is known about the learning behavior of new entrepreneurs and the entrepreneurial process. Therefore, it is necessary to explore how entrepreneurial learning influences business performance of start-ups.

The relationship between entrepreneurial learning and firm performance has received increasing attention in recent years (Zhao et al., 2011; Tseng, 2013; Sinha et al., 2019). Most studies have documented that firms engaging in more entrepreneurial learning perform better, while several studies have found that entrepreneurial learning has no or even negative effect on venture performance under certain circumstances. These mixed findings indicate that simply examining the direct relationship between entrepreneurial learning and firm performance is incomplete. Thus, this relationship requires a wider analysis of intermediate steps between them. Entrepreneurial self-efficacy (ESE) reflects the level of an individual's confidence in entrepreneurial capability (Chen et al., 1998), and can be influenced by mastery experiences (Zhao et al., 2005). In this respect, entrepreneurial learning contributes to enhancing individuals' ESE. Yet, few studies have considered the mediating effect of ESE on entrepreneurial learning and corporate performance, leaving a “black box” in understanding. In addition, the process of entrepreneurial learning is affected by the firm environment. Nonetheless, there are little studies that test the joint impact of entrepreneurial learning and entrepreneurial orientation on ESE.

Extant research asserts that entrepreneurs encounter different challenges during each particular phase (Lewis and Churchill, 1983; Sullivan, 2000). Especially, startups are fragile and fickle during the conception and survival phases (Wani, 2018). A nascent entrepreneur, a person who initiates actions that are intended to culminate in a viable new firm (Reynolds, 1994), generally face high levels of causal ambiguity and complexity and suffers from a liability of newness (Muñoz-Bullon et al., 2015). At the same time, nascent entrepreneurs generally have less entrepreneurial experience and weaker entrepreneurial capabilities in comparison with mature entrepreneurs, leading to survival rate of new enterprises is quite low. These challenges specific to inception stage can drive nascent entrepreneurs to receive entrepreneurial learning, thereby improving their entrepreneurial skills and helping the nascent venture to successfully move toward an operating entity. Consequently, it is necessary to research the early entrepreneurial learning efforts by the nascent entrepreneur. In our study, based on social cognitive theory, we focus on nascent entrepreneurs as research objects and propose a research model that unlocks the influence of entrepreneurial learning on firm performance by examining the mediating effect of ESE. Moreover, the role of entrepreneurial orientation in moderating this relationship is explored.

To sum up, we contribute to the literature in three ways. First, this study extends the research of the early stage of new ventures by focusing on the entrepreneurial learning behavior of nascent entrepreneurs. Second, we enrich entrepreneurship literature by offering insights into how and where entrepreneurial learning take place. Our study specifies the entrepreneurial learning process by incorporating ESE as a mediator and entrepreneurial orientation as a moderator. Finally, we contribute to the entrepreneurial learning literature by putting emphasis on the importance of the entrepreneurial orientation, cultural values of firm, in changing people's initiative, and effectiveness of entrepreneurial learning on ESE.

## Theory and Hypothesis

### Entrepreneurial Learning and Firm Performance

Entrepreneurial learning involves “the complex ways in which entrepreneurs learn to adapt their role and develop new behavior in order to negotiate the management and growth of their business” (Cope, 2005). Specifically, entrepreneurial learning involves how new knowledge is created and how it is embodies and utilized (Cope, 2005). There is a consensus that knowledge is an essential source of opportunity and can positively influence new venture performance (Lattacher et al., 2021). Consequently, entrepreneurial learning always has a positive impact on entrepreneurial outcomes through helping entrepreneurs accumulate and update knowledge (Minniti and Bygrave, 2001). For example, Politis (2005) proposed that entrepreneurial learning can enhance the ability to recognize and act on opportunities, as well as handle liabilities of newness, which are generally considered to be essential for successful entrepreneurs (Politis, 2005).

Entrepreneurship research has found that entrepreneurial learning processes may take on a variety of forms, such as congenital learning, experiential learning, vicarious learning, and so on (Kolb, 1984; Huber, 1991; Cegarra-Navarro and Wensley, 2009; Sullivan et al., 2021). Congenital knowledge refers to knowledge available at the organization's birth, which is mainly from founders (Huber, 1991). Cegarra-Navarro and Wensley (2009) emphasized that congenital learning is the process of learning from organizational founders, which is able to successfully guide the firm through its early stages. Experiential learning is considered an important means of updating knowledge stock. Entrepreneurs can get a higher-level learning from critical events through deep reflection and mental models changes (Lattacher et al., 2021). Besides, entrepreneurs can also compensate for their lack of knowledge via observing the behavior of others or listening to the experiences shared by others (Mansoori, 2017). Many studies have found that vicarious learning is seen as an effective knowledge acquisition means especially in unfamiliar and uncertain fields (Holcomb et al., 2009; Casillas et al., 2015).

Given the liabilities of newness and smallness, the understanding of the learning process relating to nascent entrepreneurs is of great importance (Yusuf, 2012; Cosenz and Noto, 2018). Following the perspective of entrepreneurial learning channels, the existing research indicates entrepreneurial learning for nascent entrepreneurs mainly includes formal organizational learning, intergenerational learning, and social network learning (Wang et al., 2018; Wu, 2018; Sullivan et al., 2021; Yousaf et al., 2021). Formal organizational learning refers to entrepreneurship education or training program during school or in start-ups usually covering both business skills and entrepreneurship attitudes (Sun, 2020). It has emerged as a primary way of learning for entrepreneurs to meet the complex demands and performance associated with the changing world of work. Besides formal entrepreneurial education, vicarious learning represented by intergenerational learning and social network learning also plays an important role in entrepreneurial progress. Many studies have underlined that business family offspring have stronger entrepreneurial ability and business parents play significant roles in supporting their children's entrepreneurial learning (Edelman et al., 2016; Wang et al., 2018). In addition, social network learning is an indispensable learning channel which can help entrepreneurs gain access to key resources, information, and even political capital from colleagues, customers, suppliers, competitors, advisory agencies, support services, and so on (Davidsson and Honig, 2003; Wu et al., 2018).

Numerous studies have demonstrated that entrepreneurial learning has a positive halo effect on new venture's continuity and success (Cope, 2005; Wang, 2008). There is increasing consensus that entrepreneurial learning is critical for improving performance and creating value (Cope, 2005; Sullivan et al., 2021). Firstly, entrepreneurial learning can provide ample knowledge and skills to boost the growth of new ventures. Knowledge are major strategic resources and crucial to venture's competitive advantage in a dynamic environment (Baum et al., 2001; Harrison and Leitch, 2005). For example, Sullivan et al. (2021) argued that learning different domains activities, such as customer leaning, financial learning, technology learning, and so on, can shape individual an (initial) stock of entrepreneurial knowledge to cope with business challenge. Cope (2005) proposed that entrepreneurial learning enables entrepreneurs to access to technological know-how and gain a clearer picture of internal business needs, requirements for growth and future strategic directions. Secondly, entrepreneurial learning contributes to opportunity identification and search, and lay the path for entrepreneurs' effective decision-making. Entrepreneurs can obtain important market and policy information by learning from social network partners, which is conductive to opportunity exploration and exploitation (Ardichvili et al., 2003; Cooper et al., 2016; Xiang et al., 2017; Sullivan et al., 2021; Wu et al., 2021a). In addition, through entrepreneurial learning, entrepreneurs can accumulate their skills and abilities, shape their attitudes, and beliefs (Harvey and Evans, 1995). These improved cognitive capabilities can help firm to recognize current and potentially future opportunities and formulate an effective differentiation strategy that works to foster firm growth (Xiang et al., 2017). Accordingly, we hypothesize that:

***Hypothesis 1***. *Entrepreneurial learning is positively associated with firm performance*.

### The Mediating Effect of Entrepreneurial Self-Efficacy

According to social cognitive theory, self-efficacy is defined as an individual's belief in his or her own capability to accomplish certain tasks (Bandura and McClelland, 1977). Specifically, a person with high self-efficacy perceive himself as capable of mobilizing the motivation, cognitive resources, and courses of action to meet given situational demands (Bandura and Wood, 1989). Self-efficacy predominantly stems from performance accomplishments, vicarious experience, social persuasion, and physiological states (Bandura and McClelland, 1977). Self-efficacy is considered as a motivational mechanism that not only enables people to set higher goals but also strengthens the likelihood of goal achievement, which positively affects performance (Bandura, 1991). Bandura (2010) stressed the cornerstone role of self-efficacy in human motivation, performance accomplishments, and emotional well-being (Bandura, 2010). For instance, researchers concur with this and observe that people with high self-efficacy can create positive expectations regarding future performance and motivate themselves to strive for goals even if in undesirable circumstances (Bandura, 1997; Baumgartner et al., 2008).

Following generalized self-efficacy, entrepreneurship scholars proposed the construct of ESE, an individual's cognitive estimation of capability for successfully performing given tasks in entrepreneurship context (Chen et al., 1998). Entrepreneurial self-efficacy reflects perceived feasibility and plays a crucial role in behavioral choices and performance outcomes (McGee and Peterson, 2019; Newman et al., 2019). The existing literature shows that ESE is effective in influencing goal commitment, aspiration levels, task persistence, and work attitude (Krueger and Dickson, 1994). For example, Individuals with high ESE tend to carry out risk-taking or opportunity recognition behavior (Izquierdo and Buelens, 2011). Substantial research remains focused on the effectiveness of ESE in pursuing entrepreneurial careers and initiating a new venture (Newman et al., 2019).

There is growing evidence that entrepreneurial learning fosters ESE. In line with social cognitive theory, entrepreneurial learning provides opportunities to influence motivation and behavior through the pathways of mastery experiences, vicarious learning, social persuasion, and physiological state (Zhao et al., 2005; Newman et al., 2019). For example, enactive mastery experiences feed into persons' ESE expectancy and these can be enhanced through professional courses, business case competition, entrepreneurship training projects, simulated or real business exercises, etc. (Zhao et al., 2005; Wilson et al., 2007). Vicarious learning take place by means of observing successful role models such as prestigious entrepreneurs who are successfully managing enterprise (Zhao et al., 2005). Entrepreneurship instructors also use social persuasion to give feedback on one's abilities to cope successfully (Wilson et al., 2007). Finally, entrepreneurial learning also involves the work and lifestyle of successful entrepreneurs and teaches psychological coping strategies, which will help cope with anxiety and build self-confidence (Zhao et al., 2005).

Entrepreneurial learning can equip potential entrepreneurs with knowledge and skills, thus foster ESE level to achieve desired outcomes. First, entrepreneurial learning provides chances to advance in business tactics, which will lead to the growth of self-efficacy in individuals. Yousaf et al. (2021) argue that individuals who receive entrepreneurial education will be more confident to identify opportunities, allocate resources, and even conduct an enterprise. Second, the impact of entrepreneurial learning on entrepreneurial performance becomes effective when self-efficacy is affected. There is increasing consensus that ESE is a robust predictor of entrepreneurial intention and firm performance. Researchers find that entrepreneurs possessed higher in ESE can lead their firms to higher levels of revenue and employment growth (Baum and Locke, 2004). In sum, we argue that entrepreneurial leaning, such as formal organizational learning, intergenerational learning, social network learning, etc., enhances the self-efficacy of individuals, thereby improving their entrepreneurial performance.

The preceding analysis posits that entrepreneurial learning improves the ESE by enhancing knowledge acquisition and creation. In turn, ESE can motivate entrepreneurs to create higher performance. Thus, entrepreneurial learning is assumed to foster and enhance ESE, and also is expected to improve venture performance outcomes through its effect on ESE. Therefore:

***Hypothesis 2***. *Entrepreneurial self-efficacy will mediate the impact of entrepreneurial learning on firm performance*.

### The Moderating Effect of Entrepreneurial Orientation

Although we hypothesize that entrepreneurial learning contributes to prompting ESE of nascent entrepreneurs, certain conditions may augment or constrain those effects. Organizational environment, such as entrepreneurial orientation, is an important contingency in shaping entrepreneurial learning's impact on nascent entrepreneur's self-efficacy.

Entrepreneurial orientation is a firm-level strategic orientation and holds a central position in the domain of entrepreneurship (Palmer et al., 2019). Entrepreneurial orientation construct initially reflects the strategic postures of established organizations (McGee and Peterson, 2019), which provides a basis for decision-making practices, managerial behavior, and entrepreneurial actions (Rauch et al., 2009). Specifically, it embodies the management-related preferences, beliefs, behaviors, and certain firm-level outcomes that corporate executives hope to express (Covin et al., 2006). There is a general consensus that the dimensionality of entrepreneurial orientation is predominantly includes innovativeness, risk-taking, and proactiveness (Miller, 1983; Covin and Slevin, 1989). Another widely-used dimensional view of entrepreneurial orientation expands competitive aggressiveness and autonomy on the basis of three core dimensions above (Lumpkin and Dess, 1996).

Previous scholars have emphasized that entrepreneurial orientation is a strong predictor of firm performance (Covin and Slevin, 1989; Zahra and Covin, 1995; Wiklund and Shepherd, 2005; Palmer et al., 2019). Firms with high entrepreneurial orientation tend to develop product-market innovations, take risks, and behave proactively in pursuit of new opportunities and growth (Miller, 1983). These characteristics help firms identify and exploit valuable opportunities and create a first-mover advantage than rivals, thus improving firm performance (McGee and Peterson, 2019). However, some scholars argue that entrepreneurial orientation as a performance enhancing predictor may fail in certain situations and it may even have a negative effect on performance (Wiklund and Shepherd, 2005; Covin et al., 2006; Moreno and Casillas, 2008). For example, Frank et al. (2010) proposed that the effect of entrepreneurial orientation on performance is affected by environmental dynamism and resource availability (Frank et al., 2010). Schepers et al. (2014) demonstrated that the positive impact of entrepreneurial orientation on financial performance decreases when the level of socioemotional wealth preservation increases in a family business context (Schepers et al., 2014).

The combination of entrepreneurial orientation and learning perspective received increasing attention in recent years (Covin et al., 2006; Wang, 2008; Real et al., 2014; Jiang et al., 2016). Extant literature has explicitly or implicitly examined firm's performance cannot be interpreted solely with entrepreneurial orientation or learning orientation (Sinha et al., 2019). Several studies have illustrated that learning can be an important mechanism through which entrepreneurial orientation is able to affect business performance (Wang, 2008; Zhao et al., 2011; Sinha et al., 2019). For instance, Wang (2008) argued that organizational learning plays an important role in entrepreneurship and learning-oriented values must be in place to maximize the effect of entrepreneurial orientation on performance. Real et al. (2014) suggested that organizational learning partially mediates the relationship between entrepreneurial orientation and performance, and fully mediates the link between learning orientation and performance. Whereas, much is known about the relationship between entrepreneurial orientation and learning at the organizational level, too little is known about individual learning behavior under entrepreneurial oriented environment. Therefore, we explore entrepreneurial learning behavior of nascent entrepreneurs in an entrepreneurial oriented context.

As mentioned above, entrepreneurs expect to improve their self-efficacy through entrepreneurial learning. High entrepreneurial orientation implies that focal firm may have an innovative, risky and proactive environment (Miller, 1983). In such context, firms encourage individuals to access to external new knowledge and resources, make continuous entrepreneurial efforts to improve skills. In other words, entrepreneurs in high entrepreneurial orientation companies, are more open to new information and more willing to engage in entrepreneurial learning activities (Jiang et al., 2016). Consequently, entrepreneurs' learning initiative and effectiveness can be increased, which can intensify the entrepreneurship capability and enhance ESE. In addition, an innovation-friendly organizational culture grant entrepreneur flexibility and freedom to exercise their creativity and champion promising ideas. In this respect, entrepreneurs' ESE can be enhanced as such environments provide greater opportunities for enactive master, vicarious learning, and lead to more positive physiological states (Cooper et al., 2016).

In sum, we propose that entrepreneurial learning's positive effect on ESE becomes more evident when focal firms have a higher entrepreneurial orientation. Thus:

***Hypothesis 3***. *The association between entrepreneurial learning and entrepreneurial self-efficacy is stronger in firms with high entrepreneurial orientation*.

**Figure 1:**
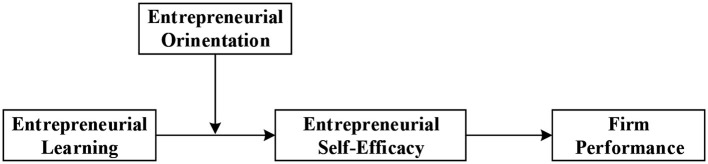
Conception framework of this study.

Based on the foregoing hypotheses, conception model is presented in Figure 1.

## Materials and Methods

### Data and Sample

To analyze the influence of entrepreneurial learning on early-stage entrepreneurship performance, we focus our study on nascent entrepreneurs. A nascent entrepreneur is a person who seriously attempts to transform business ideas into a viable new firm (Reynolds, 1994; Cassar and Craig, 2009). With the rapid economic growth and the development of entrepreneurial education in China, an increasing body of college graduates follows the trend of starting up a new venture and has become new entrants in the market. Based on Tian et al. (2018), college students who have graduated from college within the past 3 years belong to the start-up stage. Also, to encourage college students to start their own businesses, the Chinese government has issued college students venture interest free loans, which apply to university students or individuals graduated from universities for no more than 2 years. Consequently, given that national situation in China, this paper adopts a purposeful and convenient sampling (Robson, 1993), and selects young entrepreneurs who just graduated from universities within 1–2 years as the research object.

We mainly collected data from Zhejiang Province of China, which has a developed private economy and is a hotbed of entrepreneurship and innovation. As one of the most progressive provinces in the economic reform, Zhejiang Province has a relatively high entrepreneurship dynamics, which contributes to a large number of small and medium enterprises and technology start-ups in the local region (Wang et al., 2019; Zhou and Li, 2020). As stated in the Hengda Institute (2020), Zhejiang Province ranks second in the country in terms of the number of newly established market entities per 10,000 people. What's more, Zhejiang Province attaches importance to innovation and entrepreneurship education (Wu and Chen, 2015) and the construction of entrepreneurial service platforms (Chen and Pan, 2019), so that college students' start-up rates of Zhejiang rank among the top in China (Lyu et al., 2021). Therefore, we selected nascent entrepreneurs of Zhejiang Province in China as the research sample.

Universities, science parks and business incubators are believed to provide an effective vehicle to foster the creation of start-up firms (Phan et al., 2005; Ratinho and Henriques, 2010). To obtain data from multiple sources, a total of 600 questionnaires were randomly distributed to individuals from universities, science parks, and incubators in Zhejiang Province of China. The respondents were requested to fill out and return the questionnaires on site. After deleting observations with missing data, we obtain a final sample totalling 411 observations with a response rate of about 68.5%.

As shown in Table 1, the sample consist of 275 (66.9%) male entrepreneurs and 136 (33.1%) female entrepreneurs, similar to the gender distribution among young start-ups. Two hundred and eighty-seven respondents (69.8%) are entrepreneurs with vocational education background, and 124 respondents (30.2%) are entrepreneurs holding a bachelor degree or above. With respect to firm age, 90 respondents (21.9%) are in the preparatory stage of starting a new business, 252 respondents (61.3%) have established a business for <2 years, 69 respondents (16.8%) have established businesses for more than 2 years. With respect to the start-up investment scale, 204 respondents (49.6%) invested below 100 thousand, 169 respondents (41.1%) invested 100 thousand to 1 million, and 38 respondents (9.2%) invested more than 1 million.

**Table 1:** Description of sample.

**Gender**		
Male	275	66.9%
Female	136	33.1%
**Education Background**		
Higher vocational degree	35	8.5%
Junior college degree	252	61.3%
Bachelor degree	98	23.8%
Master degree	18	4.4%
Doctoral degree	8	1.9%
**Firm Age**		
Still in preparation period	90	21.9%
0–6 months	76	18.5%
6–12 months	110	26.8%
1–2 years	66	16.1%
More than 2 years	69	16.8%
**Firm Size (The Start-Up Investment Scale)**		
Below 100 thousand	204	49.6%
100–500 thousand	89	21.7%
500–1,000 thousand	80	19.5%
1,000–5,000 thousand	26	6.3%
Above 5,000 thousand	12	2.9%

### Measurements

The survey items were drawn from existing validated scales, and they were revised after extensive consultations with senior executives to fit the Chinese context for face validity. The measures used in this study are given in Supplementary Material. Key variables were measured on five-point Likert scales, which the range of response was 1 = strongly disagree and 5 = strongly agree.

#### Entrepreneurial Learning

Entrepreneurial learning construct was measured with its separate dimensions of formal organizational learning (six items), intergenerational learning (seven items), and social network learning (five items) using the 18 items, five-point scale developed expressly for this research. The measure of formal organizational learning comprehensively evaluates the entrepreneurship education through formal organizational learning, including course teaching, entrepreneurship training, business simulation competition, and other entrepreneurship practices. The measure of intergenerational learning evaluates the learning interactions among family members in respect to entrepreneurship knowledge, especially young entrepreneur's social learning from parents. The measure of social network learning was developed to assess the usefulness of social ties in exploring entrepreneurship knowledge and skills. The respondents rated their attitude to each statement against the five-point Likert scales, in which 1 = strongly disagree; 5 = strongly agree. To obtain the overall entrepreneurial learning, the scores of the 18 items were averaged and standardized.

#### Entrepreneurial Self-Efficacy

Based on Wilson et al. (2007), a total of six questions were designed to capture the respondents' level of ESE. The respondents were asked to self-report their confidence on their problem-solving ability, opinion/support solicitation ability, leadership, creativity, and decision-making ability. The measure of ESE consists of six items. The respondents rated their attitude to each statement against the five-point Likert scales, in which 1 = strongly disagree; 5 = strongly agree. To obtain the overall ESE, the scores of the six items were averaged and standardized.

#### Entrepreneurial Orientation

Entrepreneurial orientation was investigated with its separate dimensions of innovativeness (three items), proactiveness (three items), and risk-taking (three items) using the nine items, five-point scale proposed by Covin and Slevin (1989) and Real et al. (2014). To obtain the overall entrepreneurial orientation, the scores of the nine items were averaged and standardized. Higher overall scores on this scale indicate a more EO.

#### Firm Performance

Firm performance is a multidimensional construct in nature (Artz et al., 2003; Wu et al., 2021b), so its measurement needs to integrate different dimensions in empirical research (Wiklund and Shepherd, 2005). Past research has also obtained relative firm performance by measuring a firm's financial standing compared with competitors in its industry (Palmer et al., 2019). Hence, we measured four dimensions of performance relative to competitors in terms of market share, operating profits, sales volume, and employee growth in accordance with these past measures.

#### Control Variables

We controlled for factors that can potentially affect firm performance. In the context of the present study, founders' characteristics might relate to their ability to successfully engage in learning activities and it might relate to firm performance. Consistent with the previous literature, we controlled for gender, educational background, professional pertinence, family business background, and prior work experience. Besides individual level factors, firm level antecedents may affect entrepreneurship learning process and entrepreneurial outcomes. Following prior literature, we controlled for firm age and firm size. Since industry also affect firm performance, industry prosperity was controlled by dummy variables.

### Common Method Variance

Harman's one-factor test of all variables, including entrepreneurial learning, ESE, entrepreneurial orientation, and firm performance, was conducted to check for common method variance (Doty and Glick, 1998; Podsakoff et al., 2003). The results showed that seven factors with eigenvalues >1.0 accounted for 64.2% of the total variance, with the first factor accounting for only 25.2% of the total variance. These results show that common method bias is not an issue in the survey responses.

## Results

Before formal regression, several reliability tests were carried out on variable constructs. The Cronbach's alpha for the measures of formal organizational learning, intergenerational learning and social network learning is 0.8705, 0.9098, and 0.8581, respectively, indicating the high reliability of the measurement of these constructs. The Cronbach's alpha for the measure of ESE and entrepreneurial orientation is 0.8571 and 0.8570, showing high reliability of the measurement of ESE and entrepreneurial orientation.

The descriptive statistics and correlation matrix are shown in Table 2. The results show that the entrepreneurial learning is positively associated with ESE and firm performance. Similarly, educational background, professional pertinence, prior work experience, firm age, firm size, and industry prosperity are also positively associated with firm performance.

**Table 2:** Descriptive statistics and correlation.

**Variable**	***N***	**Mean**	**SD**	**1**	**2**	**3**	**4**	**5**	**6**	**7**	**8**	**9**	**10**	**11**	**12**
1.Gender	411	0.67	0.47	1											
2.Education background	411	2.30	0.77	−0.08	1										
3.Professional pertinence	411	2.71	0.97	0.09	0.15^*^	1									
4.Family business background	411	0.44	0.50	0.09	0.05	−0.22^*^	1								
5.Prior work experience	411	0.56	0.50	0.06	0.06	−0.12^*^	0.21^*^	1							
6.Firm age	411	2.87	1.37	0.04	0.22^*^	0.08	0	0.13^*^	1						
7.Industry prosperity	411	2.56	1.02	−0.11^*^	0.33^*^	0.18^*^	0.07	0.06	0.50^*^	1					
8.Firm size	411	1.91	1.10	−0.18^*^	0.36^*^	0.12^*^	−0.02	0.08	0.29^*^	0.50^*^	1				
9.Entrepreneurial self-efficacy	411	0	1	0.06	0.09	0.06	0.02	0.03	0.10^*^	0.18^*^	0.07	1			
10.Entrepreneurial orientation	411	0	1	−0.09	0.04	0	0.05	−0.09	0.01	0	0	−0.05	1		
11.Entrepreneurial learning	411	0	1	0.01	−0.08	0.25^*^	0	−0.04	−0.09	0.13^*^	0.10^*^	0.59^*^	−0.01	1	
12.Firm performance	411	0	1	−0.04	0.26^*^	0.14^*^	0.03	0.15^*^	0.30^*^	0.45^*^	0.34^*^	0.28^*^	0.02	0.21^*^	1

* *p < 0.05*.

### The Main Effect of Entrepreneurial Learning

According to Baron and Kenny's suggestion, hierarchical regression analysis was used to test the hypotheses. In all equations, the control variables were entered before the other independent variables to partial out their effects from the relationships of principal interest. The results are presented in Table 3.

**Table 3:** The hierarchical regression results.

	**Entrepreneurial self-efficacy**				**Firm performance**						
	**Model 1**	**Model 2**	**Model 3**	**Model 4**	**Model 5**	**Model 6**	**Model 7**	**Model 8**	**Model 9**	**Model 10**	**Model 11**
**Control variable**											
Gender	0.152	0.129	0.121	0.109	0.012	0.006	−0.019	−0.015	0.013	0.000	−0.015
	(0.108)	(0.085)	(0.085)	(0.085)	(0.094)	(0.093)	(0.092)	(0.092)	(0.093)	(0.093)	(0.092)
Education background	0.046	0.189^***^	0.191^***^	0.184^***^	0.117^*^	0.156^**^	0.108^*^	0.127^**^	0.155^**^	0.147^**^	0.121^**^
	(0.070)	(0.056)	(0.056)	(0.056)	(0.061)	(0.061)	(0.060)	(0.061)	(0.061)	(0.061)	(0.061)
Professional pertinence	0.025	−0.146^***^	−0.146^***^	−0.140^***^	0.060	0.013	0.055	0.036	0.013	0.019	0.039
	(0.054)	(0.044)	(0.044)	(0.044)	(0.047)	(0.048)	(0.046)	(0.048)	(0.048)	(0.048)	(0.048)
Family business background	0.011	−0.061	−0.054	−0.041	−0.018	−0.037	−0.020	−0.028	−0.042	−0.029	−0.023
	(0.104)	(0.082)	(0.082)	(0.082)	(0.091)	(0.090)	(0.089)	(0.089)	(0.090)	(0.089)	(0.089)
Prior work experience	0.040	0.046	0.037	0.036	0.238^***^	0.240^***^	0.230^***^	0.233^***^	0.247^***^	0.246^***^	0.241^***^
	(0.102)	(0.080)	(0.081)	(0.080)	(0.089)	(0.088)	(0.087)	(0.087)	(0.088)	(0.087)	(0.087)
Firm age	0.003	0.098^***^	0.098^***^	0.093^***^	0.050	0.076^**^	0.050	0.061^*^	0.075^**^	0.069^*^	0.056
	(0.042)	(0.034)	(0.034)	(0.033)	(0.037)	(0.037)	(0.036)	(0.037)	(0.037)	(0.036)	(0.037)
Industry prosperity	0.179^***^	0.056	0.055	0.053	0.296^***^	0.263^***^	0.260^***^	0.254^***^	0.263^***^	0.261^***^	0.253^***^
	(0.063)	(0.050)	(0.050)	(0.050)	(0.055)	(0.055)	(0.054)	(0.054)	(0.055)	(0.054)	(0.054)
Firm size	−0.026	−0.083^*^	−0.084^**^	−0.085^**^	0.105^**^	0.089^*^	0.110^**^	0.102^**^	0.090^*^	0.089^*^	0.101^**^
	(0.054)	(0.043)	(0.043)	(0.042)	(0.047)	(0.046)	(0.046)	(0.046)	(0.046)	(0.046)	(0.046)
**Independent variable**											
EL		1.108^***^	1.108^***^	1.112^***^		0.302^***^		0.130	0.303^***^	0.307^***^	0.148
		(0.070)	(0.070)	(0.070)		(0.076)		(0.096)	(0.076)	(0.076)	(0.096)
**Mediator variable**											
ESE							0.201^***^	0.156^***^			0.144^***^
							(0.042)	(0.054)			(0.054)
**Moderator variable**											
EO			−0.041	−0.061					0.033	0.012	0.021
			(0.039)	(0.040)					(0.043)	(0.043)	(0.043)
**Interaction terms**											
EL × EO				0.159^**^						0.167^**^	0.144^**^
				(0.062)						(0.068)	(0.068)
_cons	−0.715^***^	−0.371^**^	−0.368^**^	−0.339^*^	−1.657^***^	−1.563^***^	−1.513^***^	−1.505^***^	−1.565^***^	−1.534^***^	−1.486^***^
	(0.230)	(0.182)	(0.182)	(0.181)	(0.200)	(0.198)	(0.197)	(0.197)	(0.198)	(0.197)	(0.197)
N	411	411	411	411	411	411	411	411	411	411	411
R-Square	0.041	0.410	0.411	0.421	0.248	0.276	0.288	0.291	0.278	0.288	0.301

*EL, entrepreneurial learning; ESE, entrepreneurial self-efficacy; EO, entrepreneurial orientation*.

* *p < 0.10*

** *p < 0.05*

*** *p < 0.01*.

Our first analysis explored the predictions for the relationship between entrepreneurial learning and firm performance. Model 5 is the base model, containing only the control variables. Model 6 contains results pertaining to the main effect of entrepreneurial learning on firm performance. Model 6 shows that there is a positive relationship between entrepreneurial learning and firm performance (coefficient = 0.302, *p* < 0.01) is significant and positive. These results offer evidence confirming H1, which means engaging in entrepreneurial learning contribute to enhancing firm performance.

### The Mediating Effect of Entrepreneurial Self-Efficacy

As shown in Table 3, Model 2, Model 7, and Model 8 examine the mediation effect of ESE. Model 2 suggests entrepreneurial learning has a positive impact on ESE (coefficient = 1.108, *p* < 0.01). Model 7 shows that self-efficacy is significantly and positively associated with entrepreneurial performance (coefficient = 0.201, *p* < 0.01). Model 8 shows that entrepreneurial learning is not significant, while ESE is significantly and positively associated with firm performance (coefficient = 0.156, *p* < 0.01). Taken together, these results support H2, which means ESE mediates positive relationship between entrepreneurial learning and corporate performance.

### The Moderating Effect of Entrepreneurial Orientation

Table 3 also reports the results of testing H3, which predict positive moderation of entrepreneurial orientation on the entrepreneurial learning and ESE. Models 4 and Model 11 contain the interaction terms around which the hypotheses were offered. The results of Model 4 suggest that the interactive effect of entrepreneurial learning and entrepreneurial orientation on ESE is significant and positive (coefficient = 0.159, *p* < 0.05). In Model 11, the result shows that the interaction term is still significant and positive after adding all variables (coefficient = 0.144, *p* < 0.05). Hence, the results support H3 that entrepreneurial orientation strengthens the positive relationship between entrepreneurial learning and ESE.

## Discussion

### Theoretical Contributions

First, this study extends the entrepreneurial learning research to the early stage of new ventures by focusing on nascent entrepreneurs' learning behavior. Most of the previous research has focused on organizational learning and knowledge management of large companies (Zhao et al., 2011; Real et al., 2014). However, entrepreneurial learning in the context of nascent entrepreneurship has rarely been studied. Extant research has primarily examined the impacts of entrepreneurial learning on entrepreneurial intentions (Wilson et al., 2007; Hu et al., 2018; Shi et al., 2019), while little attention has been paid to understanding its impacts on established firms' outcomes. College graduates as new market entrants play an increasing role in the entrepreneurial field. This study considers college graduates entrepreneurs as the main subject, which contributes to the understanding of how nascent entrepreneurs' entrepreneurial learning influences the outcomes of the nascent entrepreneurship process.

Second, this study contributes to the entrepreneurial learning-firm performance research stream focusing on the intermediate links. It is a widely held belief that entrepreneurial learning increases firm performance, but we know little about mediation effect in such a link (Real et al., 2014; Chen and Pan, 2019). Specifically, our study explains this relationship by an indirect effect through ESE. Our results show that ESE fully mediates the relationship between entrepreneurial learning and firm performance. It takes into account the fact that entrepreneurial learning can prompt entrepreneur's confidence in copying with entrepreneurial tasks and stimulate firm to create a better performance. Therefore, entrepreneurial learning can be viewed as a long investment on individuals' ESE and firm performance. These findings specify how entrepreneurial learning take place, which enrich entrepreneurship learning literature.

Finally, we contribute to the entrepreneurial learning literature by identifying the significance of the entrepreneurial orientation. Prior research has explored the relationship between entrepreneurial orientation and learning at the organizational level (Wang, 2008; Zhao et al., 2011; Real et al., 2014), but there are few studies on individual learning behavior under entrepreneurial oriented environment. Along this line, we explore entrepreneurial learning behavior of nascent entrepreneurs in an entrepreneurial oriented context. Regarding the influence of entrepreneurial orientation as a moderating variable, entrepreneurial learning seems to have a greater impact on ESE in firms with high entrepreneurial orientation. High entrepreneurial orientation firm constantly seek out new opportunities, positively engage in product-market innovation, bravely undertakes somewhat risky investment. Such characteristics are associated with improved willingness and effectiveness of entrepreneurial learning on ESE. In this respect, the positive relationship between entrepreneurial learning and ESE will be strengthened if firms have a high entrepreneurial orientation. Our results advance previous research by noting that entrepreneurial orientation as an import entrepreneurial cultural environment of firms can affect the relationship between entrepreneurial learning on ESE.

### Managerial Implications

Two key managerial implications are evident in our research findings. First, much importance should be attached to entrepreneurial learning if we want to cultivate individuals' ESE or prompt business performance. Specifically, entrepreneurs should make full use of entrepreneurial learning channels, such as formal organizational learning, intergenerational learning, and social network learning, to enhance their entrepreneurial competencies and achievements. Second, it is necessary to create a favorable entrepreneurial oriented cultural environment. Entrepreneurial orientation is a managerial attitude that must be supported by certain organizational conditions that facilitate learning. Entrepreneurs as the leader of the enterprise, should positively cultivate entrepreneurial orientation and create a desirable cultural environment for entrepreneurial learning.

### Limitations and Future Research

This study has several limitations that future should address. First, we acknowledge that the geographical limitation of data collection would affect the generalizability of the results, although Zhejiang Province plays an essential role in the entrepreneurship development in China. Future research could be conducted with large-scale samples from more coastal and inland provinces so that the sample can be more representative for the Chinese context. Besides, future research is expected to utilize a cross-national sample to further explore the generalizability of the model, because Chinese entrepreneurship environments are different from those in a Western context.

Second, one limitation of this study is that we only focus on nascent entrepreneurs with firms that were still in business. For those nascent entrepreneurs that had discontinued entrepreneurship, it is difficult to collect data from them. In addition, we explored the relationship between entrepreneurial learning and firm performance in a limited time, but we did not include a time sequence in our cross-sectional data. Thus, future studies should collect panel data to trace the entrepreneurship process of these nascent entrepreneurs and longitudinal research is need to clarify the effect over time of entrepreneurial learning.

Third, future work could link ESE to overconfidence and over-optimism. Although we found ESE has a positive effect on firm performance, but we know there exists “the too much of a good thing effect,” which means their relationship may have negative or curvilinear effects in some circumstances. Finally, more research is needed on the firm characteristics construct such as strategic orientation and assess these factors which led to the development of entrepreneurial learning process.

## Data Availability Statement

The raw data supporting the conclusions of this article will be made available by the authors, without undue reservation.

## Ethics Statement

Ethical review and approval were not required for the study on human participants in accordance with the local legislation and institutional requirements. The patients/participants provided their written informed consent to participate in this study.

## Author Contributions

All authors listed have made a substantial, direct and intellectual contribution to the work, and approved it for publication.

## Conflict of Interest

The authors declare that the research was conducted in the absence of any commercial or financial relationships that could be construed as a potential conflict of interest.

## Publisher's Note

All claims expressed in this article are solely those of the authors and do not necessarily represent those of their affiliated organizations, or those of the publisher, the editors and the reviewers. Any product that may be evaluated in this article, or claim that may be made by its manufacturer, is not guaranteed or endorsed by the publisher.

## References

[B1] Ardichvili A. Cardozo R. Ray S. (2003). A theory of entrepreneurial opportunity identification and development. J. Bus. Ventur. 18, 105–123. 10.1016/S0883-9026(01)00068-4

[B2] Artz K. W. Norman P. M. Hatfield D. E. (2003). Firm performance: a longitudinal study of R&D, patents, and product innovation. Acad. Manag. Proc. 2003, B1–B6. 10.5465/ambpp.2003.13792264

[B3] Bandura A. (1991). Social cognitive theory of self-regulation. Organ. Behav. Hum. Decis. Process. 50, 248–287. 10.1016/0749-5978(91)90022-L

[B4] Bandura A. (1997). Self-Efficacy: The Exercise of Control. New York, NY: Freedom and Company.

[B5] Bandura A. (2010). Self-efficacy, in The Corsini Encyclopedia of Psychology, eds Weiner I. B. Craighead W. E. (Hoboken, NJ: John Wiley and Sons, Inc.), 1–3.

[B6] Bandura A. McClelland D. C. (1977). Social Learning Theory. Englewood Cliffs, NJ: Prentice Hall.

[B7] Bandura A. Wood R. (1989). Effect of perceived controllability and performance standards on self-regulation of complex decision making. J. Pers. Soc. Psychol. 56, 805–814. 10.1037/0022-3514.56.5.805 2724068

[B8] Baum J. R. Locke E. A. (2004). The relationship of entrepreneurial traits, skill, and motivation to subsequent venture growth. J. Appl. Psychol. 89, 587–598. 10.1037/0021-9010.89.4.587 15327346

[B9] Baum J. R. Locke E. A. Smith K. G. (2001). A multidimensional model of venture growth. Acad. Manage. J. 44, 292–303. 10.5465/3069456

[B10] Baumgartner H. Pieters R. Bagozzi R. P. (2008). Future-oriented emotions: conceptualization and behavioral effects. Eur. J. Soc. Psychol. 38, 685–696. 10.1002/ejsp.467

[B11] Casillas J. C. Barbero J. L. Sapienza H. J. (2015). Knowledge acquisition, learning, and the initial pace of internationalization. Int. Bus. Rev. 24, 102–114. 10.1016/j.ibusrev.2014.06.005

[B12] Cassar G. Craig J. (2009). An investigation of hindsight bias in nascent venture activity. J. Bus. Ventur. 24, 149–164. 10.1016/j.jbusvent.2008.02.003

[B13] Cegarra-Navarro J. G. Wensley A. K. P. (2009). Congenital learning in the Spanish telecommunication industry. J. Bus. Ventur. 24, 533–543. 10.1016/j.jbusvent.2008.05.009

[B14] Chen C. C. Greene P. G. Crick A. (1998). Does entrepreneurial self-efficacy distinguish entrepreneurs from managers? J. Bus. Ventur. 13, 295–316. 10.1016/s0883-9026(97)00029-3

[B15] Chen Y. N. Pan J. Y. (2019). Do entrepreneurs' developmental job challenges enhance venture performance in emerging industries? A mediated moderation model of entrepreneurial action learning and entrepreneurial experience. Front. Psychol. 10:1371. 10.3389/fpsyg.2019.01371 31244744 PMC6579820

[B16] Cooper D. Peake W. Watson W. (2016). Seizing opportunities: the moderating role of managerial characteristics on the relationship between opportunity-seeking and innovation efficacy in small businesses. J. Small Bus. Manage. 54, 1038–1058. 10.1111/jsbm.12228

[B17] Cope J. (2005). Toward a dynamic learning perspective of entrepreneurship. Entrepr. Theory Pract. 29, 373–397. 10.1111/j.1540-6520.2005.00090.x

[B18] Cosenz F. Noto G. (2018). Fostering entrepreneurial learning processes through Dynamic Start-up business model simulators. Int. J. Manag. Educ. 16, 468-482. 10.1016/j.ijme.2018.08.003

[B19] Covin J. G. Green K. M. Slevin D. P. (2006). Strategic process effects on the entrepreneurial orientation–sales growth rate relationship. Entrepren. Theory Pract. 30, 57–81. 10.1111/j.1540-6520.2006.00110.x

[B20] Covin J. G. Slevin D. P. (1989). Strategic management of small firms in hostile and benign environments. Strateg. Manage. J. 10, 75–87. 10.1002/smj.4250100107

[B21] Davidsson P. Honig B. (2003). The role of social and human capital among nascent entrepreneurs. J. Bus. Ventur. 18, 301–331. 10.1016/S0883-9026(02)00097-6

[B22] Doty D. H. Glick W. H. (1998). Common methods bias: does common methods variance really bias results? Organ. Res. Methods 1, 374–406. 10.1177/109442819814002

[B23] Edelman L. F. Manolova T. Shirokova G. Tsukanova T. (2016). The impact of family support on young entrepreneurs' start-up activities. J. Bus. Ventur. 31, 428–448. 10.1016/j.jbusvent.2016.04.003

[B24] Feng B. Chen M. (2020). The impact of entrepreneurial passion on psychology and behavior of entrepreneurs. Front. Psychol. 11:1733. 10.3389/fpsyg.2020.01733 32793066 PMC7385187

[B25] Frank H. Kessler A. Fink M. (2010). Entrepreneurial orientation and business performance — a replication study. Schmalenb. Bus. Rev. 62, 175–198. 10.1007/BF03396804

[B26] Harrison R. T. Leitch C. M. (2005). Entrepreneurial learning: researching the interface between learning and the entrepreneurial context. Entrepren. Theory Pract. 29, 351–371. 10.1111/j.1540-6520.2005.00089.x

[B27] Harvey M. Evans R. (1995). Strategic windows in the entrepreneurial process. J. Bus. Ventur. 10, 331–347. 10.1016/0883-9026(95)00037-9

[B28] Hengda Institute (2020). The China Youth Entrepreneurship Development Report. Available online at: http://www.yee.org.cn/qywtzgg/202010/P020201030628330192124.pdf

[B29] Holcomb T. R. Ireland R. D. Holmes R. M. Hitt M. A. (2009). Architecture of entrepreneurial learning: exploring the link among heuristics, knowledge, and action. Entrepren. Theory Pract. 33, 167–192. 10.1111/j.1540-6520.2008.00285.x

[B30] Hu R. Wang L. Zhang W. Bin P. (2018). Creativity, proactive personality, and entrepreneurial intention: the role of entrepreneurial alertness. Front. Psychol. 9:951. 10.3389/fpsyg.2018.00951 29962985 PMC6011088

[B31] Huber G. P. (1991). Organizational learning: the contributing processes and the literatures. Organ. Sci. 2, 88–115. 10.1287/orsc.2.1.88

[B32] Izquierdo E. Buelens M. (2011). Competing models of entrepreneurial intentions: the influence of entrepreneurial self-efficacy and attitudes. In. J. Entrepren. Small Bus. 13, 75–91. 10.1504/IJESB.2011.040417

[B33] Jawula S. (2021). Effective Leadership Strategies to Sustain Small Businesses' Operations Beyond 5 Years. 10723, Walden University.

[B34] Jiang X. Yang Y. Pei Y.-L. Wang G. (2016). Entrepreneurial orientation, strategic alliances, and firm performance: inside the black box. Long Range Plann. 49, 103–116. 10.1016/j.lrp.2014.09.003

[B35] Jin C. Wu B. Hu Y. (2021). Family business internationalization in paradox: effects of socioemotional wealth and entrepreneurial spirit. Front. Psychol. 12:1095. 10.3389/fpsyg.2021.667615 33967925 PMC8100041

[B36] Klimas P. Czakon W. Kraus S. Kailer N. Maalaoui A. (2021). Entrepreneurial failure: a synthesis and conceptual framework of its effects. Eur. Manage. Rev. 18, 167–182. 10.1111/emre.12426

[B37] Kolb D. A. (1984). Experience as the Source of Learning and Development. Upper Sadle River, NJ: Prentice Hall.

[B38] Krueger N. Jr. Dickson P. R. (1994). How believing in ourselves increases risk taking: perceived self-efficacy and opportunity recognition. Decis. Sci. 25, 385–400. 10.1111/j.1540-5915.1994.tb00810.x

[B39] Lattacher W. Gregori P. Holzmann P. Schwarz E. J. (2021). Knowledge spillover in entrepreneurial emergence: a learning perspective. Technol. Forecast. Soc. Change 166:120660. 10.1016/j.techfore.2021.120660

[B40] Lewis V. L. Churchill N. C. (1983). The Five Stages of Small Business Growth. University of Illinois at Urbana-Champaign's Academy for Entrepreneurial Leadership Historical Research Reference in Entrepreneurship.

[B41] Liu X. Lin C. Zhao G. Zhao D. (2019). Research on the effects of entrepreneurial education and entrepreneurial self-efficacy on college students' entrepreneurial intention. Front. Psychol. 10:869. 10.3389/fpsyg.2019.00869 31068862 PMC6491517

[B42] Lumpkin G. T. Dess G. G. (1996). Clarifying the entrepreneurial orientation construct and linking it to performance. Acad. Manage. Rev. 21, 135–172. 10.5465/amr.1996.9602161568

[B43] Lyu J. Shepherd D. M. Lee K. (2021). Teaching entrepreneurship in China: culture matters. Int. J. Entrepren. Behav. Res. 27, 1285–1310. 10.1108/IJEBR-09-2020-0653

[B44] Mansoori Y. (2017). Enacting the lean startup methodology. Int. J. Entrepren. Behav. Res. 23, 812–838. 10.1108/IJEBR-06-2016-0195

[B45] McGee J. E. Peterson M. (2019). The long-term impact of entrepreneurial self-efficacy and entrepreneurial orientation on venture performance. J. Small Bus. Manage. 57, 720–737. 10.1111/jsbm.12324

[B46] Miller D. (1983). The correlates of entrepreneurship in three types of firms. Manage. Sci. 29, 770–791. 10.1287/mnsc.29.7.770

[B47] Minniti M. Bygrave W. (2001). A dynamic model of entrepreneurial learning. Entrepren. Theory Pract. 25, 5–16. 10.1177/104225870102500301

[B48] Moreno A. M. Casillas J. C. (2008). Entrepreneurial orientation and growth of SMEs: a causal model. Entrepren. Theory Pract. 32, 507–528. 10.1111/j.1540-6520.2008.00238.x

[B49] Muñoz-Bullon F. Sanchez-Bueno M. J. Vos-Saz A. (2015). Startup team contributions and new firm creation: the role of founding team experience. Entrepren. Region. Dev. 27, 80–105. 10.1080/08985626.2014.999719

[B50] Newman A. Obschonka M. Schwarz S. Cohen M. Nielsen I. (2019). Entrepreneurial self-efficacy: a systematic review of the literature on its theoretical foundations, measurement, antecedents, and outcomes, and an agenda for future research. J. Vocat. Behav. 110, 403–419. 10.1016/j.jvb.2018.05.012

[B51] Palmer C. Niemand T. Stöckmann C. Kraus S. Kailer N. (2019). The interplay of entrepreneurial orientation and psychological traits in explaining firm performance. J. Bus. Res. 94, 183–194. 10.1016/j.jbusres.2017.10.005

[B52] Parker S. C. (2013). Do serial entrepreneurs run successively better-performing businesses? J. Bus. Ventur. 28, 652–666. 10.1016/j.jbusvent.2012.08.001

[B53] Phan P. H. Siegel D. S. Wright M. (2005). Science parks and incubators: observations, synthesis and future research. J. Bus. Ventur. 20, 165–182. 10.1016/j.jbusvent.2003.12.001

[B54] Podsakoff P. M. MacKenzie S. B. Lee J. Y. Podsakoff N. P. (2003). Common method biases in behavioral research: a critical review of the literature and recommended remedies. J. Appl. Psychol. 88, 879–903. 10.1037/0021-9010.88.5.879 14516251

[B55] Politis D. (2005). The process of entrepreneurial learning: a conceptual framework. Entrepren. Theory Pract. 29, 399–424. 10.1111/j.1540-6520.2005.00091.x

[B56] Ratinho T. Henriques E. (2010). The role of science parks and business incubators in converging countries: evidence from Portugal. Technovation 30, 278–290. 10.1016/j.technovation.2009.09.002

[B57] Rauch A. Wiklund J. Lumpkin G. T. Frese M. (2009). Entrepreneurial orientation and business performance: an assessment of past research and suggestions for the future. Entrepren. Theory Pract. 33, 761–787. 10.1111/j.1540-6520.2009.00308.x

[B58] Real J. C. Roldán J. L. Leal A. (2014). From entrepreneurial orientation and learning orientation to business performance: analysing the mediating role of organizational learning and the moderating effects of organizational size. Brit. J. Manage. 25, 186–208. 10.1111/j.1467-8551.2012.00848.x

[B59] Reynolds P. (1994). Autonomous firm dynamics and economic growth in the United States, 1986–1990. Reg. Stud. 28, 429–442. 10.1080/00343409412331348376

[B60] Robson C. (1993). Real World Research: A Resource for Social Scientists and Practitioner-Researchers. Oxford: Blackwell Publishers Inc.

[B61] Schepers J. Voordeckers W. Steijvers T. Laveren E. (2014). The entrepreneurial orientation–performance relationship in private family firms: the moderating role of socioemotional wealth. Small Bus. Econ. 43, 39–55. 10.1007/s11187-013-9533-5

[B62] Shi L. Yao X. Wu W. (2019). Perceived University support, entrepreneurial self-efficacy, heterogeneous entrepreneurial intentions in entrepreneurship education: the moderating role of the Chinese sense of face. https://www.emerald.com/insight/publication/issn/2053-4604 J. Entrepren. Emerg. Econ. 12, 205–230. 10.1108/JEEE-04-2019-0040

[B63] Sinha K. K. Steel P. Saunders C. Dewald J. R. (2019). Synergistic impacts of entrepreneurial and learning orientations on performance: a meta-analysis. Acad. Manage. Proc. 2019:11822. 10.5465/ambpp.2019.156

[B64] Sullivan D. M. Marvel M. R. Wolfe M. T. (2021). With a little help from my friends? How learning activities and network ties impact performance for high tech startups in incubators. Technovation 101:102209. 10.1016/j.technovation.2020.102209

[B65] Sullivan R. (2000). Entrepreneurial learning and mentoring. Int. J. Entrepren. Behav. Res. 6, 160–175. 10.1108/13552550010346587

[B66] Sun X. M. (2020). Exploration and practice of “internet plus maker education” University innovative entrepreneurship education model from the perspective of positive psychology. Front. Psychol. 11:891. 10.3389/fpsyg.2020.00891 32581900 PMC7294890

[B67] Tian X. Z. Wu Y. Y. Wang Y. Y. (2018). Career calling of nascent entrepreneurs in China: structure and measurement. Soc. Behav. Pers. 46, 695–704. 10.2224/sbp.6656

[B68] Tseng C. C. (2013). Connecting self-directed learning with entrepreneurial learning to entrepreneurial performance. Int. J. Entrepren. Behav. Res. 19, 425–446. 10.1108/IJEBR-08-2011-0086

[B69] Wang C. L. (2008). Entrepreneurial orientation, learning orientation, and firm performance. Entrepren. Theory Pract. 32, 635–657. 10.1111/j.1540-6520.2008.00246.x

[B70] Wang D. Wang L. Chen L. (2018). Unlocking the influence of family business exposure on entrepreneurial intentions. Int. Entrepren. Manage. J. 14, 951–974. 10.1007/s11365-017-0475-2

[B71] Wang Z. X. Shou M. H. Wang S. Dai R. N. Wang K. Q. (2019). An empirical study on the key factors of intelligent upgrade of small and medium-sized enterprises in China. Sustainability 11, 1–16. 10.3390/su11030619

[B72] Wani K. C. (2018). Strategies to Sustain Small Businesses Beyond 5 Years. Walden University.

[B73] Wiklund J. Shepherd D. (2005). Entrepreneurial orientation and small business performance: a configurational approach. J. Bus. Ventur. 20, 71–91. 10.1016/j.jbusvent.2004.01.001

[B74] Wilson F. Kickul J. Marlino D. (2007). Gender, entrepreneurial self–efficacy, and entrepreneurial career intentions: implications for entrepreneurship education. Entrepren. Theory Pract. 31, 387–406. 10.1111/j.1540-6520.2007.00179.x

[B75] Wu B. (2018). From individual social capital to collective social capital: empirical evidence from inter-firm financing trust network. J. Chin. Sociol. 5:18. 10.1186/s40711-018-0088-3

[B76] Wu B. Jin C. Monfort A. Hua D. (2021a). Generous charity to preserve green image? Exploring linkage between strategic donations and environmental misconduct. J. Bus. Res. 131, 839–850. 10.1016/j.jbusres.2020.10.040

[B77] Wu B. Liang H. Chan S. (2021b). Political connections, industry entry choice and performance volatility: evidence from China. Emerg. Mark. Finance Trade 1–10. 10.1080/1540496X.2021.1904878

[B78] Wu B. Liang H. Shen Y. (2018). Political connection, ownership, and post-crisis industrial upgrading investment: evidence from China. Emerg. Mark. Finance Trade 54, 2651-2668. 10.1080/1540496X.2018.1491400

[B79] Wu C. Chen Y. (2015). Reformation of “three in one” entrepreneurship education model, in Proceedings of the 2015 International Conference on Social Science, Education Management and Sports Education, ed Chen L. (Beijing), 1482–1485.

[B80] Xiang Y. Chen X. Mei L. Chen J. (2017). Influence of social networks, opportunity identification on the performance of internet entrepreneurship: the evidence of Zhejiang Province in China, in 2017 Portland International Conference on Management of Engineering and Technology (PICMET) (Portland, OR), 1–8.

[B81] Yousaf U. Ali S. A. Ahmed M. Usman B. Sameer I. (2021). From entrepreneurial education to entrepreneurial intention: a sequential mediation of self-efficacy and entrepreneurial attitude. Int. J. Innov. Sci. 13, 364–380. 10.1108/IJIS-09-2020-0133

[B82] Yusuf J.-E. (2012). A tale of two exits: nascent entrepreneur learning activities and disengagement from start-up. Small Bus. Econ. 39, 783–799. 10.1007/s11187-011-9361-4

[B83] Zahra S. A. Covin J. G. (1995). Contextual influences on the corporate entrepreneurship-performance relationship: a longitudinal analysis. J. Bus. Ventur. 10, 43–58. 10.1016/0883-9026(94)00004-E

[B84] Zhao H. Seibert S. E. Hills G. E. (2005). The mediating role of self-efficacy in the development of entrepreneurial intentions. J. Appl. Psychol. 90, 1265–1272. 10.1037/0021-9010.90.6.1265 16316279

[B85] Zhao Y. Li Y. Lee S. H. Chen L. B. (2011). Entrepreneurial orientation, organizational learning, and performance: evidence from China. Entrepren. Theory Pract. 35, 293–317. 10.1111/j.1540-6520.2009.00359.x

[B86] Zhou H. Li L. (2020). The impact of supply chain practices and quality management on firm performance: evidence from China's small and medium manufacturing enterprises. Int. J. Prod. Econ. 230:107816. 10.1016/j.ijpe.2020.107816

