# Supplementary material for: Entrepreneurial Learning, Self-Efficacy, and Firm Performance: Exploring Moderating Effect of Entrepreneurial Orientation
Source: Front Psychol. 2021 Aug 26;12:731628. doi: 10.3389/fpsyg.2021.731628 (PMC8426342; doi:10.3389/fpsyg.2021.731628)
Supplement: Supplementary file 1 [file Table_1.pdf]

Supplementary Material

1 Supplementary Table

Measuring Items for Theoretical Constructs in Questionnaire

Theoretical constructs	Measuring Items	Statements about theoretical constructs in questionnaire
Intergenerational learning	Item IGL_1	Your family members often invite you to participate their business activities
	Item IGL_2	Your family members often discuss with you about their business operation or works.
	Item IGL_3	When your family members are talking about their works or business operations, you often listen and participate on the side
	Item IGL_4	Your family member often encourages you to familiar with their business operation and works.
	Item IGL_5	Your family member often invites you to participate relevant commercial conference or parties.
	Item IGL_6	Your family member often shares with you their experience about professional work or business operations.
	Item IGL_7	You learned many relevant knowledge about entrepreneurship and business operation from you family members and their firms.
Social Network Learning	Item SNL_1	You meet and communicate with professional persons from investment companies or banks and learn some business skills by utilizing your social network.
	Item SNL_2	You meet and communicate with personnel from local government or public organizations and learn some business skills by utilizing your social network.
	Item SNL_3	You meet and communicate with personnel from professional training or consulting organizations and learn some business skills by utilizing your social network.
	Item SNL_4	You meet and communicate with potential partners, consumers, suppliers etc. and learn some business knowledge by utilizing your social network.
	Item SNL_5	You often discuss or share opinions with your daily friends or acquaintances by utilizing your social network.
Formal organizational learning	Item FOL_1	What I leaned in professional courses includes lots of knowledge that useful for my entrepreneurship
	Item FOL_2	The entrepreneurship training program organized by local government or school provides lots of knowledge that useful for my entrepreneurship
	Item FOL_3	What I leaned from video, on-line course, books etc. in school or during starting business provides lots of knowledge that useful for my entrepreneurship
	Item FOL_4	The entrepreneurship competition and relevant activities I participated provides lots of knowledge that useful for my entrepreneurship

	Item FOL_5	The community activities and other practical activities I participated provides lots of knowledge that useful for my entrepreneurship
	Item FOL_6	Part-time jobs and similar experiences I take before start my own business provides lots of knowledge that useful for my entrepreneurship
Entrepreneurial Self-efficacy	Item ESE_1	Comparing with my classmate or teammate, I am always more capable to solve problems
	Item ESE_2	Comparing with my classmate or teammate, I am always more capable to persuade others to accept my opinions
	Item ESE_3	Comparing with my classmate or teammate, I am always more capable to manage financial resources
	Item ESE_4	Comparing with my classmate or teammate, I am always more creative and have more innovative ideas
	Item ESE_5	Comparing with my classmate or teammate, I am always more capable to play the leaderships in our team
	Item ESE_6	Comparing with my classmate or teammate, I am always more capable to make decisions quickly
Entrepreneurial Orientation	Item EO_1	In general, our team tends to operate innovative products with leading technology
	Item EO_2	Our team has launched many new products (services) in the last year
	Item EO_3	The products (services) launched by our team are significantly more innovative than the existing products in the industry
	Item EO_4	In general, our team tend to choose projects with high risks and high returns
	Item EO_5	Our team tends to achieve company goals through bold and rapid actions based on the external business environment.
	Item EO_6	Our team tends to adopt a bold and positive attitude to grasp potential opportunities when making decisions in the face of uncertainty.
	Item EO_7	In peer competition, our team usually takes action first, and then competitors follow up or respond
	Item EO_8	Our team tends to be a market leader, always first in introducing new products, services, or technologies
	Item EO_9	The top management team often review the industry development trend, take the lead in grasping opportunities and act early to respond to changes
